# Transcriptome Changes Associated with Boron Deficiency in Leaves of Two Citrus Scion-Rootstock Combinations

**DOI:** 10.3389/fpls.2017.00317

**Published:** 2017-03-14

**Authors:** Xiao Liu, Jia-Wei Zhang, Ling-Xia Guo, Yong-Zhong Liu, Long-Fei Jin, Syed Bilal Hussain, Wei Du, Zhao Deng, Shu-Ang Peng

**Affiliations:** Key Laboratory of Horticultural Plant Biology, Ministry of Education, College of Horticulture and Forestry Sciences, Huazhong Agricultural UniversityWuhan, China

**Keywords:** citrus, graft, boron deficiency, RNA-seq, DEG

## Abstract

Boron (B) deficiency stress is frequently observed in citrus orchards and causes considerable loss of productivity and fruit quality. Carrizo citrange (Cc) has been reported as a rootstock more tolerant to B deficiency than Trifoliate orange (To). The ‘Newhall’ navel orange (Ns) performed better when grafted onto Cc (Ns/Cc) than when grafted onto To (Ns/To) under long-term B deficiency. The present study confirmed that Ns/Cc had higher boron content, leaf fresh weight, lower leaf chlorosis and stronger photosynthesis ability than Ns/To. Moreover, B-deficiency significantly reduced the chlorophyll and carotenoid content in Ns/To. The content of total soluble sugar and lignin were dramatically increased and the expression levels of photosynthesis-related genes were substantially down-regulated in Ns/To by B-deficient treatment. B-deficiency also strongly induced expression levels of chlorophyll decomposition-related genes, glucose synthesis-related genes and lignin synthesis-related genes, and significantly inhibited the expression of carotenoid synthesis-related genes in Ns/To. Overall, these findings suggested that the influence of To on the scion of Ns was worse than that of Cc due to differently regulating these metabolic pathways under the long term of B-deficiency. The transcriptome analysis provided further information for understanding the mechanism of the different responses of scion-rootstock combinations to B-deficiency stress.

## Introduction

Boron (B) plays important roles in numerous metabolic and physiological processes of higher plants, including sugar transport, cell wall synthesis and lignification, cell wall structure maintenance, carbohydrate metabolism, RNA metabolism, respiration, indole acetic acid metabolism, phenol metabolism, and membrane transport (Blevins and Lukaszewski, 1998; Brown et al., 2002; Bolaños et al., 2004; Marschner, 2012). B deficiency is a widespread problem that leads to enlargement of root tips, accumulation of carbohydrates and starch, inhibition of photosynthetic capacity, deformation of leaves and hypertrophy of petioles (Han et al., 2008; Marschner, 2012; Yang et al., 2013; Wang et al., 2015).

Different responses to B deficiency among genotypes within species have long been recognized in many crops. For instance, Geng et al. (2003) revealed that cotton cultivars tolerant to B deficiency had greater uptake rates and improved B utilization compared with cultivar sensitive to B deficiency. In rape, the seedling height and the rapeseed quality of B-efficient plants were slightly affected by B deficiency stress compared with that of B-inefficient plants (Geng et al., 1998). Moreover, comparison of seven citrus rootstocks under B-deficient conditions showed that Carrizo citrange (Cc) was the most tolerant genotype while Trifoliate orange (To) was the most sensitive genotype, according to the parameters of dry mass, leaf area, seedling height and the content of mineral nutrients (Zhou et al., 2013).

The rootstock not only supplies water, mineral nutrients and some hormones to the scion, but it also regulates the metabolism of shoots through the exchange of genetic information (Ken et al., 2009; Jensen et al., 2010; Goldschmidt, 2014). In eggplant, scions grafted onto cold-intolerant rootstocks had higher chilling injury indexes and electrolyte leakage rates than those grafted onto cold-tolerant rootstocks (Gao et al., 2016). Huang et al. (2009) found that under NaCl stress cucumber plants grafted onto salt tolerant rootstocks had an overall improvement in fruit quality including an increase of soluble sugar, titratable acidity and vitamin C. Thus, the performance of different rootstocks can directly influence the growth of scions. However, most research has focused on physiological changes. The transcriptional changes of scions as influenced by rootstocks are still rarely reported.

In China, B deficiency stress exists in many agricultural crops, including citrus, and affects crop productivity and quality (Han et al., 2008; Zhou et al., 2013). To and Cc are widely used rootstocks in China and other citrus cultivation regions of the world. As mentioned above, Cc is more tolerant to B deficiency than To (Zhou et al., 2013). Previous studies also indicated that ‘Newhall’ navel orange (Ns), one of the major scion cultivars in China, grafted onto Cc exhibited better performance and higher B utilization than that grafted onto To (Sheng et al., 2010; Wang et al., 2014). Thus, in this study, we performed genome-wide transcriptome profiling of leaves of Ns grafted onto the To and Cc rootstocks. The different scion-rootstock combinations were treated with B-sufficient (25 μM) or B-deficient (0 μM) solution for 180 days. The purpose of this study was to get insight into the molecular mechanisms of the effect of different rootstocks on the same scion in citrus under B-deficient conditions. A genome-wide analysis of gene expression profiling after a long-term B deficiency treatment was performed in the experiment. Our results yielded numbers of differentially expressed genes (DEGs) related to the phenotype of the scion grafted onto the different rootstocks.

## Materials and Methods

### Plant Materials and Treatments

‘Newhall’ navel orange [Ns, *Citrus sinensis* (L.) Osb. cv. Newhall] was selected as a scion and grafted onto Trifoliate orange [To, *Poncirus trifoliata* (L.) Raf.] and Carrizo citrange [Cc, *C. sinensis* (L.) Osb. × *P. trifoliata* (L.) Raf.]. Then, there were two experimental groups, namely, Ns grafted onto Cc (Ns/Cc) and Ns grafted onto To (Ns/To).

The experiment was conducted in a greenhouse at Huazhong Agricultural University, Wuhan, China. The plants were clearly washed with tap water and transplanted into 10-L black plastic pots filled with B-free quartz sand and perlite (1:1, v/v) medium. Then, the plants were supplied with a modified B-free 1/4 strength Hoagland’s nutrient solution for several days, until three to five new leaves expanded in the scions. Thereafter, the plants were irrigated with a modified Hoagland’s nutrient solution with a B concentration of 25 μM (B-sufficient) or 0 μM (B-deficient). This experiment lasted for 180 days. In the end, the Ns leaves from five plants (five replicates) in each treatment were separately collected. All these samples were immediately frozen in liquid nitrogen and stored at -80°C.

### Boron Content, Photosynthetic Parameters, Chlorophyll Content, Carotenoid Content, Soluble Sugar Content, and Lignin Content Measurements

For B content analysis, 0.30 g of each sample was dry ashed in a muffle furnace at 500°C for 6 h, then dissolved in 15 mL HCl (1 mol⋅L^-1^). B concentration was determined by inductively coupled plasma–atomic emission spectrometry (ICP-AES, IRIS-Advan type, Thermo, USA) (Krejc Ová and Černohorský, 2003). There were five replicates per treatment.

The net photosynthetic rate (Pn, μmol CO_2_ m^-2^⋅s^-1^), stomatal conductance of water vapor (Gs, mmol H_2_O m^-2^⋅s^-1^), and sub-stomatal CO_2_ concentration [Ci, μmol CO_2_ mol^-1^ (air)] were measured at steady state under light saturation (1200 μmol m^-2^⋅s^-1^) and 400 ppm CO_2_ with an LI-6400 (LI-COR, Lincoln, NE, USA). One measurement per plant was performed on a fully expanded mature leaf (third or fourth leaf from the shoot apex). Five plants were measured for each treatment.

The leaf chlorophyll and carotenoid content of the plants were extracted and measured based on the method of Wei et al. (2013). Fresh leaf tissue (0.5 g) was homogenized in 25 mL of 80% acetone and kept for 15 min in the dark. The samples were then spun at 5,000 rpm for 15 min. The absorbance of the supernatant was measured at 663, 644, and 470 nm using a spectrophotometer (Shimadzu UV-1800, Japan). Total chlorophyll and carotenoid concentration were calculated in terms of fresh weight (FW). There were six replicates per treatment.

The total soluble sugar was determined based on the method given by Qayyum et al. (2011). Fresh leaves (0.1 g) were added with 5 mL of 80% ethanol to test tubes, placed in a water bath and heated for 1 h at 80°C. Then, 1 mL of the sample extract was placed into another set of test tubes and mixed with 1 mL each of 9% phenol and distilled water, subsequently standing at room temperature for 1 h. Finally, 5 mL of sulfuric acid was added and the whole mixture was vortexed. Absorbance was read at 490 nm on a UV spectrophotometer (Shimadzu UV-1800, Japan). Ethanol (80%) was used as a sample blank. Each treatment was set with three replicates.

The lignin content was determined by the lignin test kit (Comin Biotechnology Co.) according to its manual. There were three replicates per treatment (two disks from the same leaf per replicate).

### RNA Extraction, Sequencing, *De novo* Assembly and Quantifying Gene Expression

In each treatment, equal but small amount of Ns leaves from each plant were pooled together and then divided into two samples (two replicates) for RNA-Seq experiments. The total RNA of each sample was isolated using the Trizol Kit (Promega, USA) following the manufacturer’s instructions. RNA quality was verified using a 2100 Bioanalyzer (Agilent Technologies, Santa Clara, CA, USA) and checked by RNase free agarose gel electrophoresis.

The cDNA library from each sample was sequenced on the Illumina HiSeq^TM^ 4000 platform (Illumina Inc., Hayward, CA, USA) and sequencing strategy was PE150. For the library assembly, the adapter sequences and low-quality reads (base quality < 20, read length < 40 bp) were removed from the raw reads. The high-quality reads were mapped to the sweet orange reference genome sequence^1^ by Tophat2 (Kim et al., 2013). Gene expression levels were calculated as reads per kilobase of exon model per million mapped reads (RPKM). For genes with more than one alternative transcript, the longest transcript was selected to calculate the RPKM. The sets of DEGs were identified using edgeR with a |Log_2_(fold-change)| > 1.0 and an FDR ≤ 0.05. Clustered profiles with *p*-value ≤ 0.05 were considered to be significantly expressed. Then, the DEGs in each profile were subjected to gene ontology (GO) classifications by using WEGO (Ye et al., 2006).

### qRT-PCR Validation and Expression Analysis

Quantitative real-time PCR (qRT-PCR) was performed with a Roche LightCycler 480 Real-Time System (Roche, Switzerland) to examine expression patterns of 14 selected unigenes. Specific primers for the DEGs were designed by Primer 3.0 (Koressaar and Remm, 2007) and are listed in Supplementary Table S1. The citrus β-actin was used as an internal control to normalize the expression levels of the target genes among different samples. The qRT-PCR was conducted with three biological repetitions. Each biological repetition ran two technical repetitions. All the qRT-PCR reactions were arranged on a 384-well plate. qRT-PCR was performed in a 10 μL reaction volume that contained 5 μL Thunderbird TM SYBR qPCR Mix (TOYOBO, JAPAN), 1 μL of cDNA, 1 μM gene-specific primers and 3 μL ddH_2_O. Reactions started with an initial incubation at 50°C for 2 min and 95°C for 10 min and were then subjected to 40 cycles of 95°C for 15 s and 60°C for 60 s (Liu et al., 2014). The Livak (Livak and Schmittgen, 2001) method was employed to calculate gene relative expression levels.

### Statistical Analysis

The data were evaluated by Duncan’s multiple test in the ANOVA program of SAS (SAS Institute, Cary, NC, USA). Differences were considered significant at *P* < 0.05.

## Results

### Leaf Symptoms, Fresh Weight and Boron Content

**FIGURE 1:**
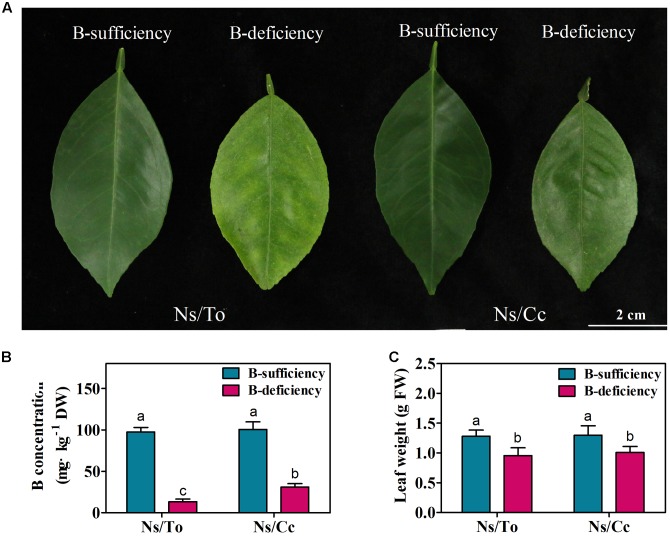
**Effect of boron deficiency on the phenotype** **(A)**, fresh weight **(B)**, and boron content **(C)** of different citrus scion-rootstock combinations. Bars with different letters indicate significant differences (*p* < 0.05) between different combinations and different treatments. Ns refers to ‘Newhall’ navel orange scion. To refers to trifoliate orange rootstock. Cc refers to Carrizo citrange rootstock.

After 180 days of B-deficient treatment, the mature leaves of Ns/To showed more severe chlorosis than those of Ns/Cc (**Figure 1A**). Moreover, compared to in the control, the B content (13.41 mg⋅kg^-1^) and fresh weight (0.95 g) showed 81.40 and 25.42% reductions in Ns/To, respectively (**Figures 1B,C**); in Ns/Cc the B content (30.91 mg⋅kg^-1^) and fresh weight (1.01 g) showed 69.24 and 22.30% reductions, respectively, compared to in the control (**Figures 1B,C**).

### Overview of the RNA Sequencing and Analysis of Differentially Expressed Genes

The transcriptome changes induced by long-term B-deficient treatment in Ns leaves were investigated through RNA-Seq analysis (**Table 1**). More than 40 million reads were generated per sample. Of these reads, the Q30 percentage (sequencing error rate < 1%) was over 94%, and GC content was approximately 45% for the libraries. Among all the libraries, 66.41–70.68% of unique reads were mapped to the sweet orange genome.

**Table 1:** Statistics of sequencing data of the four libraries.

Sample ID	Total read	GC content (%)	Q30 ratio (%)	Unique mapped reads	Multiple mapped reads	Mapping ratio
To-BS	47316886	44.49%	94.08%	32729974 (69.17%)	715504 (1.51%)	70.68%
To-BD	43408404	44.50%	94.06%	29500685 (67.96%)	581268 (1.34%)	69.30%
Cc-BS	48813366	45.15%	94.16%	33542025 (68.71%)	667288 (1.37%)	70.08%
Cc-BD	40304126	44.40%	94.02%	26222084 (65.06%)	544326 (1.35%)	66.41%


*To refers to trifoliate orange rootstock, Cc refers to Carrizo citrange rootstock. BS refers to Boron sufficiency, BD refers to Boron deficiency.*

**FIGURE 2:**
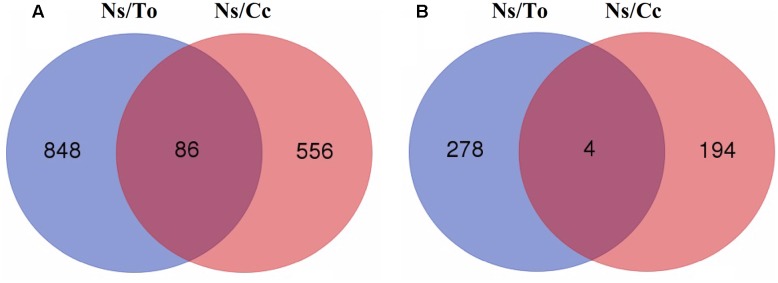
**Venn diagrams showing number of genes significantly up-regulated** **(A)** or down-regulated **(B)** in leaves with the boron-deficient treatment for 180 days. Ns refers to ‘Newhall’ navel orange scion. To refers to trifoliate orange rootstock. Cc refers to Carrizo citrange rootstock.

More than 1900 genes were significantly induced during B deficiency stress, which indicates that dramatic changes in gene expression were induced in Ns/To and Ns/Cc. A total of 934 genes in Ns/To and 642 genes in Ns/Cc were significantly up-regulated (**Figure 2A**). Comparison of these two datasets showed that 86 genes overlapped between Ns/To and Ns/Cc (**Figure 2A**). On the other hand, genes significantly down-regulated in Ns/Cc and Ns/To numbered 198 and 282, respectively. However, only four genes overlapped between them (**Figure 2B**).

**FIGURE 3:**
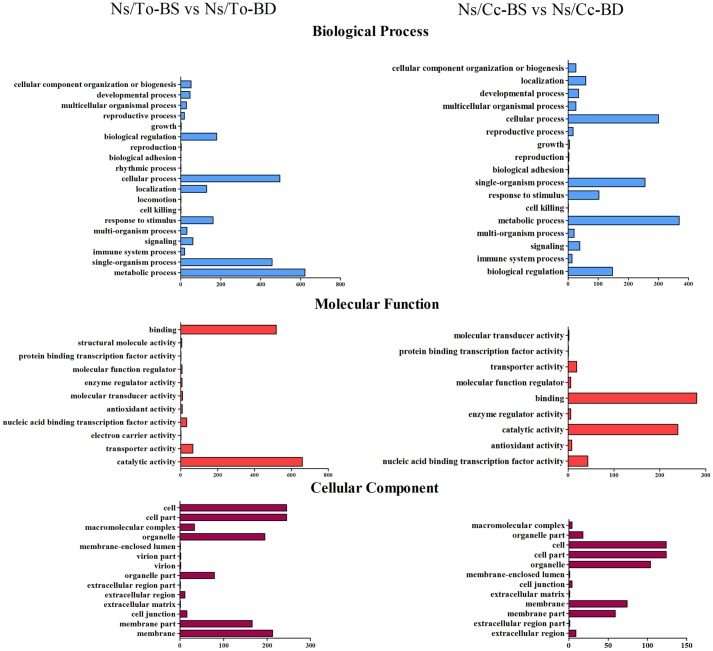
**Gene Ontology (GO) classification of differentially expressed genes (DEGs).** Ns refers to ‘Newhall’ navel orange scion. To refers to trifoliate orange rootstock. Cc refers to Carrizo citrange rootstock.

In GO annotation, long-term B deficiency had a significant effect on 19 biological processes, 11 molecular functions, and 14 cell component metabolic pathways of Ns/To (**Figure 3**). In detail, the B deficiency mainly affected the following biological processes: metabolic processes, single-organism processes, cellular processes, biological regulation and response to stimuli; the affected molecular functions included binding and catalytic activity; the cellular components affected included cells, cell parts, organelles, membranes and membrane parts. In Ns/Cc, there were only 17 biological processes, 9 molecular functions and 12 cell component metabolic pathways that were significantly induced (**Figure 3**). Specifically, metabolic processes, single-organism processes, cellular processes and biological regulation were mainly affected among the biological processes. The main changes of molecular function and cell components were similar to those of Ns/To.

To confirm their authenticity, 14 DEGs were randomly selected to analyze their expression profiles by qRT-PCR. The results of qRT-PCR analysis showed that the expression profiles of 14 DEGs were similar to those obtained through high-throughput sequencing (Supplementary Table S2). These results confirmed the reliability of the genome-wide transcriptome profiling analysis.

### Response of Photosynthesis Activity and Expression of Some Genes to B Deficiency

**FIGURE 4:**
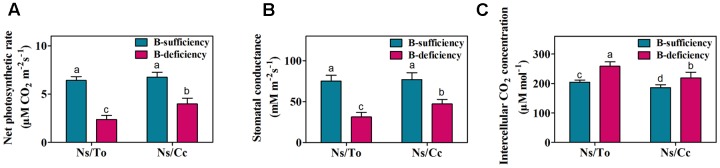
**Effect of boron deficiency on the net photosynthetic rate** **(A)**, stomatal conductance **(B)**, sub-stomatal CO_2_ concentration **(C)** of different citrus scion-rootstock combinations. Bars with different letters indicate significant differences (*p* < 0.05) between different combinations and different treatments. Ns refers to ‘Newhall’ navel orange scion. To refers to trifoliate orange rootstock. Cc refers to Carrizo citrange rootstock.

Under the B-deficient condition, the Pn and Gs of plant leaves were significantly affected. The Pn of Ns/To and Ns/Cc declined 62.97 and 41.16%, respectively (**Figure 4A**); the decline of Gs was 58.10 and 38.74%, respectively (**Figure 4B**). In contrast, the Ci of Ns/To and Ns/Cc increased 20.95 and 14.82%, respectively, with significant difference (**Figure 4C**).

Six genes involved in photosynthesis were identified as being regulated by B-deficient stress (**Table 2**). These genes included Ferredoxin-NADP reductase (Cs1g25510), ATP synthase gamma chain (Cs2g03080), PsbQ-like protein 2 (Cs4g12280), Photosystem II reaction center PSB28 protein (Cs5g29040), PSI reaction center subunit II (Cs5g31180) and Photosystem II core complex protein (Cs7g09900). The six genes were significantly down-regulated in Ns/To while only slightly down-regulated in Ns/Cc.

**Table 2:** List of boron-deficiency-responsive genes involved in photosynthesis metabolism.

Gene ID	Putative function	Log_2_^fold^ ^change^	
		
		Ns/To	Ns/Cc
**Photosynthesis**			
Cs1g25510	Ferredoxin-NADP reductase, FNR	**-1.02**	-0.34
Cs2g03080	ATP synthase gamma chain, ATPC	**-1.17**	-0.30
Cs4g12280	PsbQ-like protein 2, PQL2	**-1.24**	-0.53
Cs5g29040	Photosystem II reaction center PSB28 protein	**-1.01**	-0.61
Cs5g31180	PSI reaction center subunit II, PSAD	**-1.16**	-0.26
Cs7g09900	Photosystem II core complex proteins, PSBY	**-1.07**	-0.41


*Ns refers to ‘Newhall’ navel orange scion, To refers to trifoliate orange rootstock, Cc refers to Carrizo citrange rootstock. Significant differences in relative level are shown in bold.*

### Response of Chlorophyll and Carotenoid Content and Their Metabolism-Related Gene Expression to B Deficiency

**FIGURE 5:**
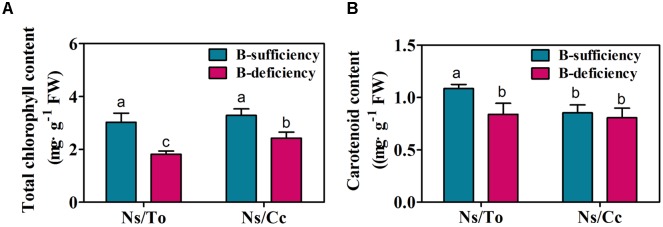
**Effect of boron deficiency on the chlorophyll** **(A)** and carotenoid content **(B)** of different citrus scion-rootstock combinations. Bars with different letters indicate significant differences (*p* < 0.05) between different combinations and different treatments. Ns refers to ‘Newhall’ navel orange scion. To refers to trifoliate orange rootstock. Cc refers to Carrizo citrange rootstock.

Under the B-deficient condition, the chlorophyll content declined significantly and carotenoid content increased significantly. The reduction of chlorophyll content was 39.91 and 26.17% in Ns/To and Ns/Cc, respectively (**Figure 5A**); the carotenoid content in Ns/To and Ns/Cc decreased 22.71 and 5.60%, respectively (**Figure 5B**).

Analysis of DEGs found that two chlorophyllase protein genes (Cs5g30790 and Cs5g16830) were significantly up-regulated in Ns/To and Ns/Cc, respectively, under B-deficient stress (**Table 3**). Moreover, the expression levels of two carotenoid synthesis genes (Cs5g03200 and Cs4g14850) were significantly decreased in Ns/To, whereas they were not significantly influenced in Ns/Cc (**Table 3**).

**Table 3:** List of boron deficiency responsive genes involved in chlorophyll and carotenoid metabolism.

Gene ID	Putative function	Log_2_^fold^ ^change^	
		
		Ns/To	Ns/Cc
**Chlorophyll metabolism**			
Cs5g30790	Chlorophyllase, CLH	**4.71**	0.29
Cs5g16830	Chlorophyllase, CLH	0.38	**1.41**
**Carotenoid metabolism**			
Cs5g03200	lycopene epsilon-cyclase, CrtR-b	**-5.51**	**-**0.34
Cs4g14850	β-carotene 3-hydroxylase, CrtL-e	**-1.34**	**-**0.52


*Ns refers to ‘Newhall’ navel orange scion, To refers to trifoliate orange rootstock, Cc refers to Carrizo citrange rootstock. Significant differences in relative level are shown in bold.*

### Response of Sugar Content and Its Metabolism-Related Gene Expression to B Deficiency

**FIGURE 6:**
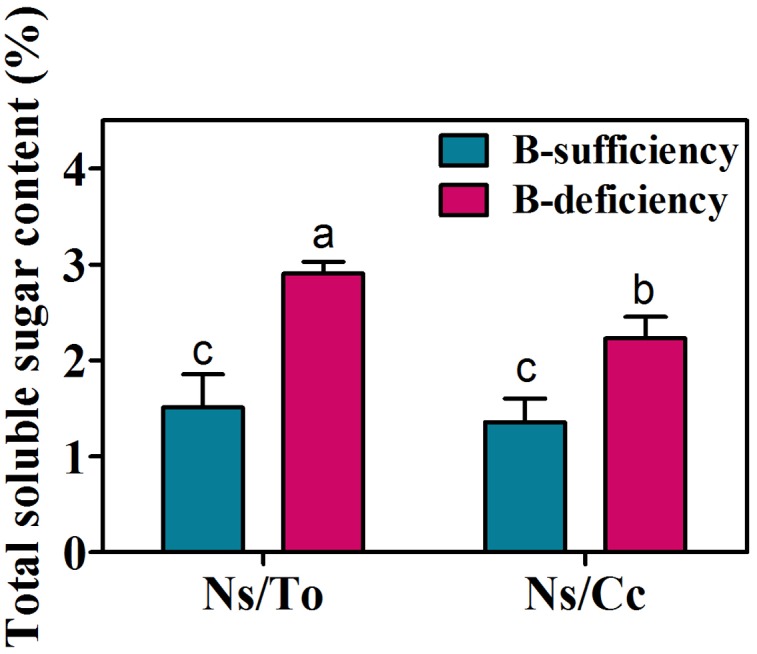
**Effect of boron deficiency on the total soluble sugar content of different citrus scion-rootstock combinations.** Bars with different letters indicate significant differences (*p* < 0.05) between different combinations and different treatments. Ns refers to ‘Newhall’ navel orange scion. To refers to trifoliate orange rootstock. Cc refers to Carrizo citrange rootstock.

B deficiency significantly increased the total sugar content in the leaves of the two scion-rootstock combinations. In detail, the total sugar content in Ns/To and Ns/Cc increased 92.27 and 64.37%, respectively (**Figure 6**).

The expression levels of three glucose synthesis genes (Cs4g07840, Cs9g14600, Cs5g01775) were significantly increased in Ns/To under B-deficient stress, whereas they were not significantly influenced in Ns/Cc (**Table 4**).

**Table 4:** List of boron deficiency responsive genes involved in sugar metabolism.

Gene ID	Putative function	Log_2_^fold^ ^change^	
		
		Ns/To	Ns/Cc
**Sugar metabolism**			
Cs4g07840	Raffinose synthase, SIP	**1.71**	0.26
Cs9g14600	Trehalose-phosphate phosphatase, TPS	**2.12**	-0.58
Cs5g01775	Trehalase, TREH	**2.17**	0.07


*Ns refers to ‘Newhall’ navel orange scion, To refers to trifoliate orange rootstock, Cc refers to Carrizo citrange rootstock. Significant differences in relative level are shown in bold.*

### Response of Lignin Content and Lignin Synthesis-Related Gene Expression to B Deficiency

**FIGURE 7:**
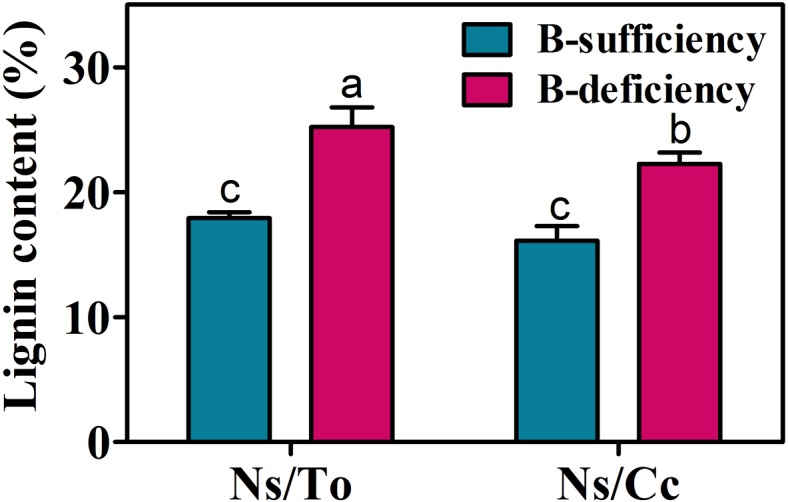
**Effect of boron deficiency on the lignin content of different citrus scion-rootstock combinations.** Bars with different letters indicate significant differences (*p* < 0.05) between different combinations and different treatments. Ns refers to ‘Newhall’ navel orange scion. To refers to trifoliate orange rootstock. Cc refers to Carrizo citrange rootstock.

The lignin contents in Ns/To and Ns/Cc were significantly increased by B-deficient treatment. The lignin contents increased 40.55 and 38.18% in Ns/To and Ns/Cc, respectively (**Figure 7**).

The expression levels of lignin synthesis-related genes, including one phenylalanine ammonia-lyase gene (Cs6g11950), two cinnamyl-alcohol dehydrogenase genes (Cs1g20580, Cs1g20610) and four peroxidase genes (Cs2g25450, Cs2g28110, orange1.1t02040, orange1.1t02043), were significantly affected by B deficiency (**Table 5**). In Ns/To, the expression levels of six genes were significantly up-regulated whereas in Ns/Cc, expression levels for only three genes were significantly up-regulated.

**Table 5:** List of boron deficiency responsive genes involved in lignin synthesis.

Gene ID	Putative function	Log_2_^fold^ ^change^	
		
		Ns/To	Ns/Cc
**Lignin synthesis**			
Cs6g11950	Phenylalanine ammonia-lyase, PAL	**2.83**	**2.41**
Cs1g20580	Cinnamyl-alcohol dehydrogenase, CAD	**1.46**	0.41
Cs1g20610	Cinnamyl-alcohol dehydrogenase, CAD	**3.25**	-0.99
Cs2g28110	Peroxidase, POD	0.67	**2.33**
Orange1.1t02041	Peroxidase, POD	**2.40**	**1.52**
Orange1.1t02040	Peroxidase, POD	**2.41**	-0.56
Orange1.1t02043	Peroxidase, POD	**2.94**	-0.88


*Ns refers to ‘Newhall’ navel orange scion, To refers to trifoliate orange rootstock, Cc refers to Carrizo citrange rootstock. Significant differences in relative level are shown in bold.*

## Discussion

In citrus, Cc was reported to be a rootstock more tolerant of B deficiency than To (Zhou et al., 2013). In addition, as an important cultivar in China, Ns is hypersensitive to B deficiency (Sheng et al., 2009; Wang et al., 2014). Previous studies found that the scion leaf growth, boron content and photosynthetic parameters in Ns/Cc were less affected by low B treatments than those in Ns/To (Sheng et al., 2010; Wang et al., 2014). In the present study, the results also showed that Ns/Cc had better leaf fresh weight, boron content and photosynthetic parameters than Ns/To (**Figures 1**, **4**) during long-term B-deficient treatment. Moreover, the present study further revealed that Ns/Cc had lower total soluble sugar, lignin and carotenoid contents but higher accumulation of chlorophyll than Ns/To (**Figures 5–7**). These results further confirmed that Cc affected its scion better than To under the B-deficient condition.

A visible symptom of B-deficient trees is leaf chlorosis in young leaves (Wang et al., 2015), which is mainly due to the reduction of chlorophyll content (Han et al., 2008, 2009). The present study showed that chlorophyll content was significantly reduced by B deficiency in both rootstock-scion combinations (**Figure 5A**). However, the reduction of chlorophyll content in Ns/Cc was less than that in Ns/To (**Figure 5A**), and the expression of the chlorophyll degradation gene, *CLH*, was significantly lower in Ns/Cc than that in Ns/To (**Table 3**). It could explain why chlorosis was lighter in the leaves of Ns/Cc than Ns/To.

B deficiency not only causes leaf chlorosis but also reduces the content of carotenoids (Moustafafarag et al., 2015). Carotenoids, which include carotenes and xanthophylls, are also important photosynthetic pigments (Zarcotejada et al., 2013). Carotenoids are physiologically relevant because of their role in photosynthesis and participation in light harvesting, energy transfer, quenching and photoprotection (Frank and Cogdell, 1996; Ritz et al., 2000). Thus, leaf chlorosis and reduction of carotenoids will directly lead to a decrease in photosynthesis (Wang et al., 2015). In this study, it was found that Ns/Cc had a lower reduction of Pn and Gs than Ns/To (**Figures 4A–C**), which suggested that the photosynthesis ability of Ns/Cc was stronger than that of Ns/To. Meanwhile, the expression levels of photosynthesis-related genes were significantly down-regulated in Ns/To while they were slightly down-regulated in Ns/Cc (**Table 2**). These results suggested that B deficiency reduced photosynthesis ability through down-regulating the expression of photosynthesis-related genes. On the other hand, the carotenoid content remarkably decreased in Ns/To (**Figure 5B**). The carotenoid synthesis-related gene were also significantly down-regulated in Ns/To (**Table 3**). Together, the significant decrease in carotenoid synthesis-related gene and photosynthesis-related gene transcript levels in Ns/To at least partially explained the reason for the lower photosynthesis ability of Ns/To than that of Ns/Cc.

The decrease of photosynthesis ability is also related to the over-accumulation of carbohydrates in leaves because a large amount of carbohydrates accumulated in the leaves produces feedback inhibition of net photosynthesis (Dell and Huang, 1997; Brown et al., 2002; Wang et al., 2015). The present study found that B deficiency led to excessive accumulation of carbohydrates in scion leaves; moreover, Ns/To had higher carbohydrate content than Ns/Cc (**Figure 6**). These results further explain the reason for the lower photosynthesis ability of Ns/To than that of Ns/Cc. In addition, this study also found that the expression levels of glucose synthesis-related genes were significantly up-regulated in Ns/To while there were no significant changes in Ns/Cc (**Table 4**), suggesting that different responses to B deficiency at the transcript level is possibly the reason for the different accumulation of carbohydrates between the leaves of Ns/To and Ns/Cc.

The typical symptom of long-term B deficiency in citrus is severe suberification in mature leaves (Yang et al., 2013; Zhou et al., 2013). This suberification is mainly due to high accumulation of lignin. Previous research on citrus showed that the expression of lignin synthesis-related genes (*CsPAL*, *CsCAD*, and *CsPOD*) increased drastically more in To than in Cc under B-deficient treatments (Yang et al., 2013; Zhou et al., 2014). In this study, the expression levels of several lignin synthesis-related genes were significantly affected by B deficiency. The expression levels of six genes were significantly up-regulated in Ns/To, while the expression levels of only three genes were significantly up-regulated in Ns/Cc (**Table 5**), which partially explains why the suberification of Ns/To was more severe than that of Ns/Cc (**Figure 1A**).

**FIGURE 8:**
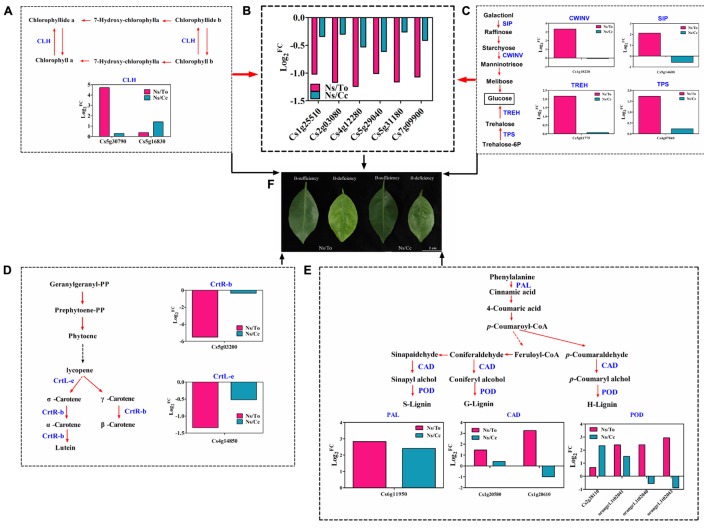
**Overview of major pathways and their expression profiles for the effect of boron deficiency on different citrus scion-rootstock combinations.** **(A–E)** Indicate the expression profiles of the DEGs involved in chlorophyll decomposition, photosynthesis, glucose synthesis, carotenoid synthesis, and lignin biosynthesis, respectively. **(F)** Phenotype of different citrus scion-rootstock combinations under 180 days of boron-deficient treatment.

In a previous study, the investigation of differences in B distribution and forms between the two combinations (Ns/To and Ns/Cc) found that the B content of buds and leaves was significantly higher in Ns/Cc plants than in Ns/To plants (Sheng et al., 2009; Wang et al., 2014). The ratio of available B was significantly lower in stems and roots of Cc-grafted than To-grafted plants under B-deficient conditions (Wang et al., 2014). In addition, a number of differentially expressed B transporter genes were identified in either Cc or To under short-term B-deficient treatments. The aquaporins genes (PIPs, TIPs) were up-regulated in Cc, but only PIP1;3 at 24 h and TIP2;2 at 12 h were induced significantly by B-deficient stress in To (Zhou et al., 2014). Transcriptomic and physiological analysis indicated that Cc had stronger B transport capacity and higher B utilization efficiency than To. Therefore, in this study, the leaves of Ns/Cc accumulated more B (**Figure 1B**). The higher B content in Ns/Cc can regulate the expression of some genes in some metabolic pathways, for example, genes involved in chlorophyll decomposition (*CsCLH*) (**Figure 8A**), photosynthesis ability (**Figure 8B**), glucose synthesis (*CsSIP*, *CsCWINV*, *CsTREH*, and *CsTPS*) (**Figure 8C**), carotenoid synthesis (*CsCrtR-b*, *CsCrtL-e*) (**Figure 8D**), and lignin synthesis (*CsPAL*, *CsPOD*, and *CsCAD*) (**Figure 8E**). Ns/Cc had fewer up-regulated expression levels of chlorophyll decomposition, sugar synthesis, and lignin synthesis genes than Ns/To, which contributed to Ns/Cc more adaptive than Ns/To to B-deficiency condition (**Figure 8F**).

## Conclusion

The present study confirmed that Ns/Cc had higher B content but lower reduction of fresh weight and photosynthesis ability than Ns/To. Moreover, this study revealed that under B-deficient treatments Ns/Cc had lower total soluble sugar and lignin but higher accumulation of chlorophyll and carotenoid contents than Ns/To. On the other hand, genes involved in photosynthesis, chlorophyll decomposition, carotenoid synthesis, sugar synthesis, and lignin metabolism were expressed differently between Ns/To and Ns/Cc. These results partially explained why Cc has a larger B deficiency tolerance influence on Ns than To. Hence, this study provided further information for understanding the molecular mechanisms of the different responses of scion-rootstock combinations in citrus to B deficiency stress.

## Data Archiving Statement

The raw data of the RNA-Seq has been uploaded to the Sequence Read Archive (SRA, https://www.ncbi.nlm.nih.gov/sra/). The accession number is SRA490165.

## Author Contributions

XL and S-AP designed the experiments. XL conceived the project, analyzed the data, and wrote the article with contributions of all the authors. J-WZ prepared experimental materials. L-XG, L-FJ, SH, WD, and ZD provided technical assistance to XL. S-AP and Y-ZL supervised and complemented the writing.

## Conflict of Interest Statement

The authors declare that the research was conducted in the absence of any commercial or financial relationships that could be construed as a potential conflict of interest.

## Footnotes

1 http://citrus.hzau.edu.cn/orange/

## References

[B1] Blevins D. G. Lukaszewski K. M. (1998). Boron in plant structure and function. *Annu. Rev. Plant Physiol. Plant Mol. Biol.* 49 481–500. 10.1146/annurev.arplant.49.1.481 15012243

[B2] Bolaños L. Lukaszewski K. Bonilla I. Blevins D. (2004). Why boron? *Plant Physiol. Biochem.* 42 907–912. 10.1016/j.plaphy.2004.11.002 15694285

[B3] Brown P. H. Bellaloui N. Wimmer M. A. Bassil E. S. Ruiz J. Hu H. (2002). Boron in plant biology. *Plant Biol.* 4 205–223. 10.1055/s-2002-25740

[B4] Dell B. Huang L. (1997). Physiological responses of plants to low boron. *Plant Soil* 193 103–120. 10.1023/A:1004264009230

[B5] Frank H. A. Cogdell R. J. (1996). Carotenoids in photosynthesis. *Photochem. Photobiol.* 63 257–264. 10.1111/j.1751-1097.1996.tb03022.x 8881328

[B6] Gao Q. H. Wu Y. Jia S. Huang S. Lu X. (2016). Effect of rootstock on the growth, photosynthetic capacity and osmotic adjustment of eggplant seedlings under chilling stress and recovery. *Pak. J. Bot.* 48 461–467.

[B7] Geng M. J. Cao X. Y. Zhu D. W. Liu W. D. Pi M. M. (1998). Effects of boron deficiency on physiological characteristics of different rape (*Brassica napus* L.) cultivars at flowering stage. *Chin. J. Oil Crop. Sci.* 20 70–73.

[B8] Geng M. J. Zhu J. H. Li-Shu W. U. Cao X. Y. Liu W. D. (2003). Effects of boron on membrane lipid peroxidation and polyamines content in leaves of two cotton cultivars with different boron efficiency at seedling stage. *Plant Nutr. Fertil. Sci.* 9 337–341.

[B9] Goldschmidt E. E. (2014). Plant grafting: new mechanisms, evolutionary implications. *Front. Plant Sci.* 5:727 10.3389/fpls.2014.00727 PMC4269114 25566298

[B10] Han S. Chen L. S. Jiang H. X. Smith B. R. Yang L. T. Xie C. Y. (2008). Boron deficiency decreases growth and photosynthesis, and increases starch and hexoses in leaves of citrus seedlings. *J. Plant Physiol.* 165 1331–1341. 10.1016/j.jplph.2007.11.002 18191499

[B11] Han S. Tang N. Jiang H. X. Yang L. T. Li Y. Chen L. S. (2009). CO2 assimilation, photosystem II photochemistry, carbohydrate metabolism and antioxidant system of citrus leaves in response to boron stress. *Plant Sci.* 176 143–153. 10.1016/j.plantsci.2008.10.004

[B12] Huang Y. Tang R. Cao Q. Bie Z. (2009). Improving the fruit yield and quality of cucumber by grafting onto the salt tolerant rootstock under NaCl stress. *Sci Hortic.* 122 26–31. 10.1016/j.scienta.2009.04.004

[B13] Jensen P. J. Makalowska I. Altman N. Fazio G. Praul C. Maximova S. N. (2010). Rootstock-regulated gene expression patterns in apple tree scions. *Tree Genet. Genomes* 6 57–72. 10.1007/s11295-009-0228-7

[B14] Ken M. Jules J. Steven S. Goldschmidt E. E. (2009). A history of grafting. *Hortic. Rev.* 35 437–493. 10.1002/9780470593776.ch9

[B15] Kim D. Pertea G. Trapnell C. Pimentel H. Kelley R. Salzberg S. (2013). TopHat2: accurate alignment of transcriptomes in the presence of insertions, deletions and gene fusions. *Genome Biol.* 14:36 10.1186/gb-2013-14-4-r36 PMC4053844 23618408

[B16] Koressaar T. Remm M. (2007). Enhancements and modifications of primer design program Primer3. *Bioinformatics* 23 1289–1291. 10.1093/bioinformatics/btm091 17379693

[B17] Krejc Ová A. Černohorský T. (2003). The determination of boron in tea and coffee by ICP–AES method. *Food Chem.* 82 303–308. 10.1016/S0308-8146(02)00566-6

[B18] Liu X. Hu X. M. Jin L. F. Shi C. Y. Liu Y. Z. Peng S. A. (2014). Identification and transcript analysis of two glutamate decarboxylase genes, CsGAD1 and CsGAD2, reveal the strong relationship between CsGAD1 and citrate utilization in citrus fruit. *Mol. Biol. Rep.* 41 6253–6262. 10.1007/s11033-014-3506-x 24976574

[B19] Livak K. J. Schmittgen T. D. (2001). Analysis of relative gene expression data using real-time quantitative PCR and the 2-ΔΔCT method. *Methods* 25 402–408. 10.1006/meth.2001.1262 11846609

[B20] Marschner H. (2012). *Mineral Nutrition of Higher Plants*, 3rd Edn San Diego, CA: Academic Press.

[B21] Moustafafarag M. Sheng F. B. Guy K. M. Yang J. Hu Z. Zhang M. F. (2015). Activated antioxidant enzymes-reduced malondialdehyde concentration, and improved mineral uptake-promoted watermelon seedlings growth under boron deficiency. *J. Plant Nutr.* 39 14 10.1080/01904167.2015.1105263

[B22] Qayyum A. Razzaq A. Ahmad M. Jenks M. A. (2011). Water stress causes differential effects on germination indices, total soluble sugar and proline content in wheat (*Triticum aestivum* L.) genotypes. *Afr. J. Biotechnol.* 10 14038–14045. 10.5897/AJB11.2220

[B23] Ritz T. Damjanoviæ A. Schulten K. Zhang J. P. Koyama Y. (2000). Efficient light harvesting through carotenoids. *Photosynth. Res.* 66 125–144. 10.1023/A:1010750332320 16228415

[B24] Sheng O. Song S. Peng S. A. Deng X. X. (2009). The effects of low boron on growth, gas exchange, boron concentration and distribution of ‘Newhall’ navel orange (*Citrus sinensis* Osb.) plants grafted on two rootstocks. *Sci. Hortic.* 121 278–283. 10.1016/j.scienta.2009.02.009

[B25] Sheng O. Zhou G. F. Wei Q. J. Peng S. A. Deng X. X. (2010). Effects of excess boron on growth, gas exchange, and boron status of four orange scion–rootstock combinations. *J. Plant Nutr. Soil Sci.* 173 469–476. 10.1002/jpln.200800273

[B26] Wang N. N. Yan T. S. Fu L. N. Zhou G. F. Liu Y. Z. Peng S. A. (2014). Differences in boron distribution and forms in four citrus scion–rootstock combinations with contrasting boron efficiency under boron-deficient conditions. *Trees* 28 1589–1598. 10.1007/s00468-014-1067-1

[B27] Wang N. N. Yang C. Q. Pan Z. Y. Liu Y. Z. Peng S. A. (2015). Boron deficiency in woody plants: various responses and tolerance mechanisms. *Front. Plant Sci.* 6:916 10.3389/fpls.2015.00916 PMC4621400 26579163

[B28] Wei Q. J. Liu Y. Z. Zhou G. F. Li Q. H. Yang C. Q. Peng S. A. (2013). Overexpression of CsCLCc, a chloride channel gene from *Poncirus trifoliata*, enhances salt tolerance in *Arabidopsis*. *Plant Mol. Biol. Rep.* 31 263–268. 10.1007/s11105-013-0592-1

[B29] Yang C. Q. Liu Y. Z. An J. C. Li S. Jin L. F. Zhou G. F. (2013). Digital gene expression analysis of corky split vein caused by boron deficiency in ‘Newhall’ navel orange (*Citrus sinensis* Osbeck) for selecting differentially expressed genes related to vascular hypertrophy. *PLoS ONE* 8:e65737 10.1371/journal.pone.0065737 PMC3673917 23755275

[B30] Ye J. Fang L. Zheng H. Zhang Y. Chen J. Zhang Z. (2006). WEGO: a web tool for plotting GO annotations. *Nucleic Acids Res.* 34 293–297. 10.1093/nar/gkl031 PMC1538768 16845012

[B31] Zarcotejada P. J. Guilléncliment M. L. Hernándezclemente R. Catalina A. González M. R. Martín P. (2013). Estimating leaf carotenoid content in vineyards using high resolution hyperspectral imagery acquired from an unmanned aerial vehicle (uav). *Agric. For. Meteorol.* 8 281–294. 10.1016/j.agrformet.2012.12.013

[B32] Zhou G. F. Liu Y. Z. Sheng O. Wei Q. J. Yang C. Q. Peng S. A. (2014). Transcription profiles of boron-deficiency-responsive genes in citrus rootstock root by suppression subtractive hybridization and cDNA microarray. *Front. Plant Sci.* 5:795 10.3389/fpls.2014.00795 PMC4309116 25674093

[B33] Zhou G. F. Peng S. A. Liu Y. Z. Wei Q. J. Han J. Islam M. Z. (2013). The physiological and nutritional responses of seven different citrus rootstock seedlings to boron deficiency. *Trees* 28 295–307. 10.1007/s00468-013-0949-y

