# Supplementary material for: Transcriptome Changes Associated with Boron Deficiency in Leaves of Two Citrus Scion-Rootstock Combinations
Source: Front Plant Sci. 2017 Mar 14;8:317. doi: 10.3389/fpls.2017.00317 (PMC5349144; doi:10.3389/fpls.2017.00317)
Supplement: Supplementary file 1 [file Table_1.DOCX]

| **Table s1. List of primers for quantitative real-time PCR** | | |
| --- | --- | --- |
| **Gene** | **Sequence (5’-3’)** | |
|  | **Forward primer** | **Reverse primer** |
| Cs7g06130 | ACCCTTTTGATTACGGCAACT | CTACAAGAAGGCTCATTTCACC |
| Cs2g17370 | TCTTATATATTACAAATCAAA | ACTCTCTTAAAGCTGCCTCTTT |
| Cs2g17810 | GGTGCAGAGATTTGGTGGCAAAG | ATGCAACGCATGGACGAAGCCGAT |
| orange1.1t04969 | ATGGATCTTCAGAAGATTAAGA | CGTCAAGATCCCTGTTATTGAT |
| orange1.1t04594 | GGCAACAAAACAACCGGCCTCGA | AAATCAAGATTTAGTGTGCTCTC |
| Cs2g30840 | ATGGCCAACGTTCAGGAGAAAGTT | AGGCCCTGCGCATAACTCAGAAA |
| Cs7g29740 | TCGTGCCATGGGGTCAACAATGG | ATCCTGGCCCTCCATCTTCTTTTC |
| Cs7g31060 | TTTGCGGGCATTTGTACGGTAAT | CATTGTCAGCAAACGTGAACC |
| Cs6g11950 | GATCAAAGAATGCAGGTCTTACC | TAGCATATTGGAAGAGGGGCGCCA |
| Cs5g01775 | ATGGAACTTGACATTGCCTCCATG | CTGGCTACTAGTTCCATTACTTATC |
| Cs5g30790 | AATGGAAACGAATCGAGGGATCC | AAACTTTTACATGAGTTGTCGTA |
| Cs4g12280 | AAAAGATATGCATTTGACCTGCT | TTCTCAAAACTTTCAAATAATCT |
| Cs5g03200 | AGGTACTACTAAAGTTGAGAAGAA | TCTGTTTGCCATGAAAATCTGT |
| orange1.1t02043 | GAGCTCACACATTTGGAAGGGCT | GAAGAAGTATTTGTTGTCAAACAA |
| Actin | CCGACCGTATGAGCAAGGAAA | TTCCTGTGGACAATGGATGGA |
