# Supplementary material for: Transcriptome Changes Associated with Boron Deficiency in Leaves of Two Citrus Scion-Rootstock Combinations
Source: Front Plant Sci. 2017 Mar 14;8:317. doi: 10.3389/fpls.2017.00317 (PMC5349144; doi:10.3389/fpls.2017.00317)
Supplement: Supplementary file 2 [file Table_2.docx]

| **Table s2. qPCR confirmation of selected transcripts identiﬁed by RNA-sequencing.** | | | | |
| --- | --- | --- | --- | --- |
| **ID** | **Log_2_^Fold change^-RNA-seq** | | **Fold change-qPCR** | |
|  | **Ns/To** | **Ns/Cc** | **Ns/To** | **Ns/Cc** |
| Cs7g06130 | 2.73 | 2.76 | 2.40 | 3.53 |
| Cs2g17370 | 2.71 | 3.65 | 3.39 | 8.26 |
| Cs2g17810 | 2.56 | 3.85 | 4.08 | 4.89 |
| orange1.1t04969 | 2.58 | 3.86 | 3.80 | 5.11 |
| orange1.1t04594 | 2.36 | 1.96 | 2.62 | 2.44 |
| Cs2g30840 | 3.27 | 14.99 | 4.74 | 17.42 |
| Cs7g29740 | 3.33 | 1.78 | 8.64 | 5.51 |
| Cs7g31060 | 3.53 | 2.15 | 4.61 | 2.95 |
| Cs6g11950 | 2.83 | 2.41 | 6.41 | 5.82 |
| Cs5g01775 | 2.17 | 0.07 | 3.71 | 1.02 |
| Cs5g30790 | 4.71 | 0.29 | 3.89 | 1.18 |
| Cs4g12280 | -1.24 | -0.53 | 0.31 | 0.78 |
| Cs5g03200 | -5.51 | -0.34 | 0.02 | 0.96 |
| orange1.1t02043 | 2.94 | -0.88 | 3.97 | 0.98 |
| Note：Ns refers to ‘Newhall’ navel orange scion, To refers to trifoliate orange rootstock, Cc refers to Carrizo citrange rootstock. | | | | |
